# Modulation of oligodendrocyte differentiation and maturation by combined biochemical and mechanical cues

**DOI:** 10.1038/srep21563

**Published:** 2016-02-16

**Authors:** Tânia Lourenço, Joana Paes de Faria, Christian A. Bippes, João Maia, José A. Lopes-da-Silva, João B. Relvas, Mário Grãos

**Affiliations:** 1 Biocant, Technology Transfer Association, Cantanhede, Portugal; 2 Centre for Neuroscience and Cell Biology (CNC), University of Coimbra, Coimbra, Portugal; 3 IBMC – Instituto de Biologia Molecular e Celular, Universidade do Porto, Porto, Portugal; 4 i3S - Instituto de Investigação e Inovação em Saúde, Universidade do Porto, Portugal; 5 Nanosurf AG, Liestal, Switzerland; 6 Chemical Engineering Department, Faculty of Science and Technology, University of Coimbra, Coimbra, Portugal; 7 QOPNA, Chemistry Department, University of Aveiro, Aveiro, Portugal

## Abstract

Extracellular matrix (ECM) proteins play a key role during oligodendrogenesis. While fibronectin (FN) is involved in the maintenance and proliferation of oligodendrocyte progenitor cells (OPCs), merosin (MN) promotes differentiation into oligodendrocytes (OLs). Mechanical properties of the ECM also seem to affect OL differentiation, hence this study aimed to clarify the impact of combined biophysical and biochemical elements during oligodendrocyte differentiation and maturation using synthetic elastic polymeric ECM-like substrates. CG-4 cells presented OPC- or OL-like morphology in response to brain-compliant substrates functionalised with FN or MN, respectively. The expression of the differentiation and maturation markers myelin basic protein — MBP — and proteolipid protein — PLP — (respectively) by primary rat oligodendrocytes was enhanced in presence of MN, but only on brain-compliant conditions, considering the distribution (MBP) or amount (PLP) of the protein. It was also observed that maturation of OLs was attained earlier (by assessing PLP expression) by cells differentiated on MN-functionalised brain-compliant substrates than on standard culture conditions. Moreover, the combination of MN and substrate compliance enhanced the maturation and morphological complexity of OLs. Considering the distinct degrees of stiffness tested ranging within those of the central nervous system, our results indicate that 6.5 kPa is the most suitable rigidity for oligodendrocyte differentiation.

Oligodendrocytes (OLs) are the myelin-forming cells of the central nervous system (CNS), wrapping axons and providing insulation to accelerate the transmission of action potentials^1^. The process of myelination occurs mostly during embryonic development and in early post-natal stages and is strictly regulated by several molecular elements, such as growth factors and hormones. While basic Fibroblast Growth Factor (bFGF) and Platelet Derived Growth Factor (PDGF) contribute to the proliferation of OL progenitors — OPCs^2^, the thyroid hormones [Triiodo-L-thyronine (T3) and Thyroxin (T4)] control the specification and differentiation of oligodendrocytes, also playing a role during the myelination of axons^3^,^4^,^5^,^6^,^7^.

The loss of OLs and consequently their myelin sheaths causes anomalous nerve transmission and neuronal cell death, as it is the case in the course of demyelinating diseases such as multiple sclerosis^8^. In demyelinating diseases, the remyelination process may be incomplete for reasons yet unclear^9^,^10^,^11^. Possible reasons are the exhaustion of OPCs or the presence of inhibitory or absence of stimulatory factors at lesioned areas which prevent the differentiation of existing progenitors^9^,^12^. Another hypothesis is the presence of a disturbed extracellular milieu, since a particular balance between extracellular adhesion and matrix rigidity seems to be required for successful myelination and remyelination to occur^13^.

The extracellular matrix (ECM) is the acellular component of organs and tissues. It is composed essentially by water, proteins and polysaccharides, providing not only physical support to cells, but also biochemical and mechanical signals necessary for tissue morphogenesis, differentiation and homeostasis (reviewed in Frantz, C. *et al*.)^14^. The biochemical composition of the extracellular matrix of the brain plays a key role during oligodendrogenesis. While the ECM proteins fibronectin (FN) or vitronectin (VN) are involved in the proliferation and maintenance of oligodendrocyte progenitors, laminin α2 (also known as merosin — MN) promotes their differentiation into mature OLs^15^,^16^,^17^,^18^,^19^,^20^. Furthermore, the extracellular matrix also comprises mechanical support. It is known that the stiffness of the extracellular milieu can modulate the fate of distinct cell types. To illustrate this idea, mesenchymal stem cells cultured on substrates compliant with the rigidity of the brain, muscle or bone were shown to display a neurogenic, myogenic or osteogenic phenotype^21^. Additionally, neural stem cells (NSCs) were shown to become specified into neuronal or glial lineages depending on substrate stiffness. Very soft platforms (100–500 Pa) promoted neuronal differentiation, while slightly stiffer ones (1,000–10,000 Pa) favoured the appearance of glial (astrocytic) cells^22^. Similarly, substrate stiffness was shown to modulate survival and proliferation of OPCs and oligodendrocyte morphology^23^,^24^.

In the present work, we sought to develop a hydrogel-based platform functionalised with extracellular matrix proteins. Combining mechanical and biochemical properties typical of the brain’s ECM allowed studying the combined effect of such factors during oligodendrocyte differentiation and maturation (unlike existing studies focusing on the effect of each component separately^16^,^18^,^23^,^24^). The results presented here indicate that the combination of mechanical and biochemical properties of the ECM present *in vivo* play a crucial role during oligodendroglial differentiation, suggesting that such factors should be taken into account when studying the biology of oligodendrocytes and in putative future clinical applications using oligodendrocyte progenitors.

## Results

### Characterization of mechanical properties of polyacrylamide hydrogels

Polyacrylamide polymers are widely used in a cell biology context due to their capacity of modelling different degrees of stiffness, which may be achieved by obtaining different crosslinking degrees by simply varying the percentage of the acrylamide (AC) and/or bis-acrylamide (BAC) monomers.

**Figure 1:**
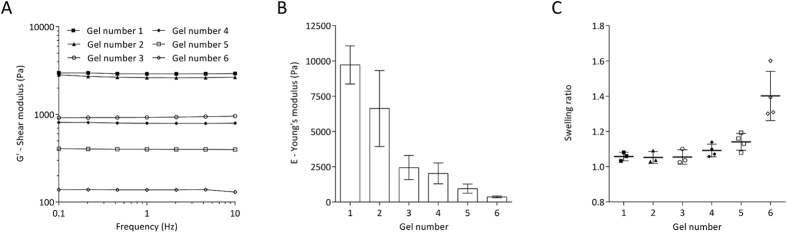
Properties of polyacrylamide hydrogels. (**A**) Representative rheological measurements of the shear storage modulus *G’* (by rheometry) of six distinct formulations of polyacrylamide hydrogels (PAHs) across a frequency sweep (0.1–10 Hz) at a constant strain (2 millistrain) and 37 °C. Mean ± SD of the Young’s modulus (**B**) or swelling ratio (**C**) of at least three independent batches of six distinct formulations of PAHs (1–6).

The mechanical properties of six formulations of polyacrylamide hydrogels (PAHs) were measured using a rheometer, by performing 0.1–10 Hz frequency sweeps (Fig. 1A). The shear storage modulus (*G′*) of all hydrogel formulations was essentially independent of the oscillatory frequency across the tested range, meaning that we are dealing with true elastic gels where any macromolecular rearrangements are very limited (Fig. 1A). The Young’s modulus (*E*) of each PAH formulation tested was calculated from the G′ values at 1 Hz as described in the “Materials and Methods” section. Increased percentage of AC and/or BAC correlated with higher stiffness of the hydrogels produced (Fig. 1B), with a range between 9720 ± 1352 Pa (gel number 1) and 362 ± 65 Pa (gel number 6), as shown in Table 1 and Fig. 1B. These formulations are therefore compliant with the range of stiffness described for central nervous system (CNS) tissue between 100 and 10,000 Pa^25^.

The hydrogels were also analysed by performing AFM measurements (Fig. S1). It could be confirmed that substrate stiffness was directly proportional to the final AC/BAC concentration used in each formulation. The absolute Young’s modulus values obtained by AFM (Fig. S1) were higher than the ones obtained by rheometry (Table 1 and Fig. 1B), although still within the kPa range. Similar differences between the nanoscale elastic modulus (AFM-nanoindentation) and the macroscopic shear modulus (assessed by rheometry) have been previously observed for a reconstituted basement membrane-like complex^26^. These discrepancies may be partially explained by different strain modes (shear or compressive, in case of rheometry or AFM, respectively) and distinct measuring time scales. Moreover, it is well known that the moduli of soft substrates (like the ones analysed here) obtained by AFM-based measurements are dependent on the conditions used. In particular, the usually unknown exact tip geometry and size as well as viscous effects of the samples that depend on the indentation velocity may lead to discrepancies^27^,^28^,^29^.

It is known that some formulations of PAHs may present high swelling ratio (SR) upon immersion in aqueous media, which could lead to different mechanical properties of the hydrogel during culture^22^ and surface creasing when SR is higher than 1.5, resulting from heterogeneous swelling due to the attachment of the substrate to a rigid support (like a glass coverslip)^30^. All PAHs produced in this study presented low SR values (lower than 1.5) — Table 1 and Fig. 1C —, hence stable in an aqueous environment. Namely, the formulations used for biological experiments (gels #1, #2 and #3) presented swelling ratio values very close to 1 [1.06 ± 0.024 (SD), 1.05 ± 0.034 (SD) and 1.06 ± 0.041 (SD), respectively].

### Progenitor- or mature-like morphology of CG-4 cells induced by fibronectin or laminin-2 was enhanced by compliant hydrogels

**Figure 2:**
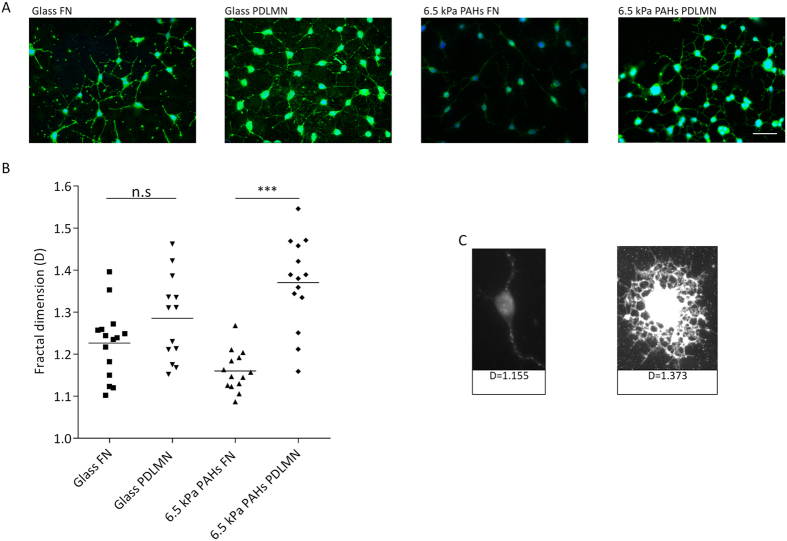
Cell morphology assessment of CG-4 cells by fractal dimension analysis. (**A**) Representative fluorescence microscopy images of oligodendroglial CG-4 cells plated on glass coverslips or 6.5 kPa polyacrylamide hydrogels (PAHs) functionalised with fibronectin (FN) or poly-D-lysine and merosin (PDLMN) and maintained in proliferation medium for 2 days. Scale bar corresponds to 50 μm. The D values (fractal dimension) are shown in (**B,C**). Values in (**B**) represent at least n = 13 cells analysed from three independent experiments and in (**C**) are depicted representative images of cells analysed in (**B**). Statistical analysis was performed by *t-test* using the software GraphPad Prism 6. Statistical comparisons were represented using connectors (n.s.: non-significant, ***p < 0.001).

Fibronectin is known to favour the maintenance of the progenitor state of oligodendrocytes^18^, while laminin-2 (also known as merosin — MN) promotes their differentiation^16^. We aimed to test whether these extracellular matrix (ECM) proteins could influence the morphology of oligodendrocytes (OLs) when coupled with substrates presenting distinct degrees of stiffness. For that, CG-4 cells (a rat CNS glial precursor cell line)^31^ were cultured in proliferation medium (to rule out the direct influence of soluble differentiation factors) for 48 h on ~6.5 kPa brain-compliant substrates (gel number 2 — Table 1) functionalised with fibronectin (FN) or poly-D-lysine/merosin (PDLMN) and, as control, on stiff (~1 GPa) glass coverslips coated with the same proteins. Cells were then stained with agglutinin (Fig. 2A,C) to assess the morphological complexity^27^ (i.e., to assess the presence of elaborate process branching) of the cells. Morphological complexity of oligodendrocytes is proportional to their differentiation state, and was quantified by fractal dimension (D) analysis, as described by others^32^,^33^. Hence, lower D values are typical of cells with progenitor phenotype presenting low process branching (Fig. 2C, left panel), while higher D values reflect a higher degree of morphological complexity (with elaborate process branching) typical of more differentiated cells^27^ (Fig. 2C, right panel). CG-4 cells showed a tendency to display a lower D value in presence of FN and higher in presence of MN (as expected) when cultured on both substrates (Fig. 2A,B). However, cells cultured on 6.5 kPa PAHs functionalised with merosin (PDLMN) presented a D value that was significantly higher (*p* < 0.0001) than that of cells cultured on the same substrate but functionalised with FN, whereas no statistically significant differences (*p* = 0.1023) were observed for cells cultured on stiff coverslips coated with the same proteins (Fig. 2B).

These results suggest that the effects of merosin and fibronectin to induce higher or lower morphological complexity (respectively) of oligodendroglial cells was enhanced by the mechanical properties of brain-compliant 6.5 kPa substrates when compared to stiff (~1 GPa) glass coverslips.

### Substrate compliance and ECM protein composition modulate the differentiation and maturation of OPCs into OLs

It was demonstrated that ECM stiffness has an impact during the differentiation of oligodendrocyte progenitor cells^23^,^24^, although the effect of the combination of ECM proteins and stiffness has not yet been reported. In the current study, the influence of substrate stiffness associated with the presence or absence of laminin-2 on the differentiation of OPCs was tested using full differentiation conditions (i.e., OPCs cultured for 3 to 5 days in presence of oligodendrocyte differentiation culture medium, as described in the ‘Materials and Methods’ section). To monitor the outcome of OPC differentiation in these distinct conditions, the expression of differentiation and maturation markers, cellular area and morphology were assessed by fluorescence microscopy. Because CG-4 cells presented low differentiation efficiency (reflected by a low percentage of MBP-positive cells — data not shown), further studies were performed using primary rat OPCs.

**Figure 3:**
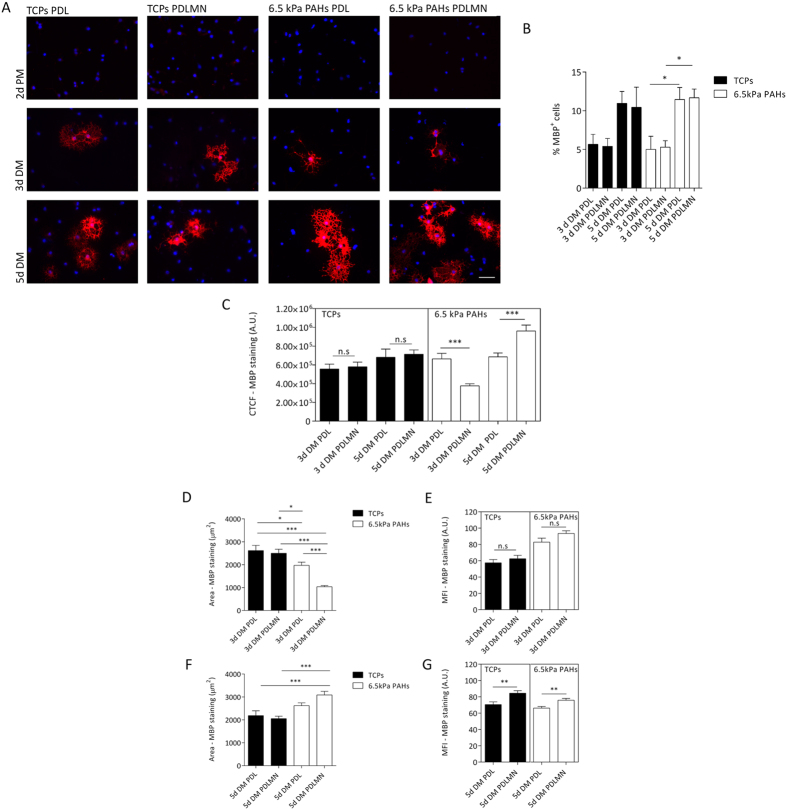
Differentiation of primary rat oligodendrocyte progenitor cells (OPCs) using distinct substrates and conditions. (**A**) Representative immunofluorescence microscopy images of primary rat oligodendrocytes stained for MBP (in red, and nuclei were counterstained with DAPI, in blue), cultured for 2 days in proliferation medium (2d PM), or for 3 or 5 days in differentiation medium (3d DM or 5d DM). Cells were maintained on TCPs or 6.5 kPa PAHs coated/functionalised with PDL or PDLMN, as indicated. Scale bars correspond to 50 μm. (**B**) The percentage of MBP-positive cells was quantified for each experimental condition (3 or 5 days in DM) and platform. Data represent mean ± SEM of at least five independent experiments. Statistical analysis was performed by two-way ANOVA followed by Bonferroni post-test. (**C**) Quantification of CTCF of MBP signal of primary rat oligodendrocytes cultured with DM for 3 or 5 days on TCPs or 6.5 kPa PAHs coated/functionalised with PDL or PDLMN, as indicated. Data represent mean ± SEM of at least 5 independent experiments. Statistical analysis was performed by *t*-test. (**D,F**) Measurement of MBP signal area of primary rat oligodendrocytes cultured with differentiation medium for 3 days (**D**) or 5 days (**F**) on TCPs or PAHs coated/functionalised with PDL or PDLMN. Data represent mean ± SEM of at least 5 independent experiments. Statistical analysis was performed by one-way ANOVA followed by Tukey’s multiple comparison test. (**E,G**) Mean fluorescence intensity (MFI) of MBP signal of oligodendrocytes cultured on TCPs or PAHs for 3 days (**E**) or for 5 days (**G**). Data represent mean ± SEM of at least 5 independent experiments. Statistical analysis was performed by *t*-test. All statistical analysis was performed using the software GraphPad Prism 6 and the statistical significant differences were represented using the connectors (n.s non-significant, *p < 0.05, **p < 0.01, ***p < 0.001).

**Figure 4:**
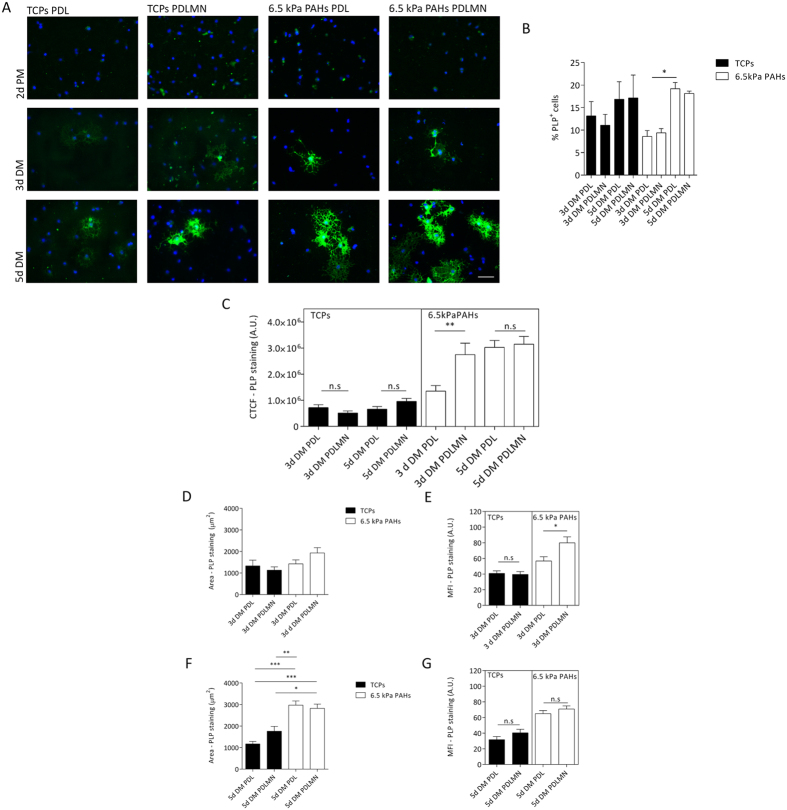
Maturation of primary rat OPCs using distinct substrates and conditions. (**A**) Representative immunofluorescence microscopy images of primary rat oligodendrocytes stained for PLP (in green, and nuclei were counterstained with DAPI, in blue), cultured for 2 days in proliferation medium (2d PM), or for 3 or 5 days in differentiation medium (3d DM or 5d DM, respectively). Cells were maintained on TCPs or 6.5 kPa PAHs coated/functionalised with PDL or PDLMN, as indicated. Scale bars correspond to 50 μm. (**B**) The percentage of PLP-positive cells was quantified for each experimental condition (3 or 5 days in DM) and platform. Data represent the mean ± SEM of at least three independent experiments. Statistical analysis was performed by two-way ANOVA followed by Bonferroni post-test. (**C**) Quantification of CTCF for PLP signal of primary rat oligodendrocytes cultured with DM for 3 or 5 days on TCPs or 6.5 kPa PAHs coated/functionalised with PDL or PDLMN. Data represent mean ± SEM of at least 3 independent experiments. (**D,F**) Measurement of the PLP signal area of primary rat oligodendrocytes cultured with DM for 3 days (**D**) or 5 days (**F**) on TCPs or PAHs coated/functionalised with PDL or PDLMN. Data represent mean ± SEM of at least 3 independent experiments. Statistical analysis was performed by one-way ANOVA followed by Tukey’s multiple comparison test. (**E,G**) Mean fluorescence intensity (MFI) of PLP signal of oligodendrocytes cultured on TCPs or PAHs for 3 days (**E**) or for 5 days (**G**). Data represent mean ± SEM of CTCF of at least 3 independent experiments. Statistical analysis was performed by *t*-test. All statistical analysis was performed using the software GraphPad Prism 6 and the statistical significant differences were represented using the connectors (n.s.: non-significant, *p < 0.05, **p < 0.01, ***p < 0.001).

OPCs were differentiated (using differentiation medium — DM) on 6.5 kPa PAHs and stiff tissue culture polystyrene plates (TCPs) functionalised or coated (respectively) with PDL or PDLMN for the indicated periods of time (Figs 3A and 4A) and the expression of MBP (oligodendrocyte differentiation marker) and PLP (OL maturation marker) were assessed by immunocytochemistry. Undifferentiated OPCs kept on proliferation medium (PM) were used as a negative control (Figs 3A and 4A).

The percentage of MBP- and PLP-positive cells was similar across substrates (Figs 3B and 4B), but increased along time of differentiation, as expected, although the only statistically significant differences between day 5 and day 3 were observed when cells were cultured on compliant 6.5 kPa substrates and not on stiff TCPs surfaces.

In order to quantify the differentiation state of oligodendrocytes between experimental conditions, the corrected total fluorescence (CTCF)^34^,^35^ per cell (from fluorescence microscopy images) using antibodies that recognize the differentiation and maturation markers MBP (Fig. 3C) and PLP (Fig. 4C), respectively, was calculated. CTCF of MBP and PLP reflects the differentiation state of oligodendrocytes^35^ by taking into account (i) the staining area of the differentiation/maturation marker^36^, correlating with the morphological differentiation state, and (ii) the mean fluorescence intensity (MFI) value^37^, that mirrors the expression level of the protein labelled per cell^38^.

After 5 days in differentiation conditions, the CTCF of MBP was statistically higher (*p* = 0.0003) in primary rat oligodendrocytes cultured on 6.5 kPa substrates functionalised with PDLMN comparing with those on PDL alone (Fig. 3C). In contrast, the CTCF for MBP of OLs cultured on TCPs in presence of the same proteins was not statistically different (*p* = 0.7407) — Fig. 3C. This indicates that the combined effect of the presence of merosin and substrate compliance (6.5 kPa) was determinant for the full differentiation of oligodendrocytes. Western-blot analysis data was in line with these results, revealing a tendency for a higher increase of MBP expression induced by laminin-2 when OPCs were differentiated on compliant substrates (6.5 kPa) compared with those maintained on stiff TCPs (Fig. S2).

Since the CTCF of MBP reflects differentiation of cells based on the combination of morphological aspects and expression level of the protein (a well-established differentiation marker), we sought to decouple the two parameters — signal area and MFI — in order to get further insight into the contribution of substrate rigidity and the presence or absence of merosin on each differentiation component. After 5 days of differentiation, MBP area was significantly higher for cells cultured on PAHs functionalised with PDLMN than on TCPs (either in presence or absence of MN), which indicates that oligodendrocytes cultured on compliant substrates in presence of merosin acquired a more mature morphological state when compared with cells differentiated on standard culture conditions (Fig. 3F). Moreover, the presence of MN also seemed to favour the expression of MBP, as observed by a statistically significant increase of the MFI of MBP at day 5, but in this case, independent of the substrate used [*p* = 0.0016 (PAHs) and *p* = 0.0017 (TCPs)] (Fig. 3G). In summary, data indicate that at day 5 of differentiation the combination of substrate compliance and merosin contributed to the morphological maturation of MBP^ + ^oligodendrocytes (Fig. 3F), whereas the presence of MN alone contributed mostly to increased expression of MBP (Fig. 3G).

Surprisingly, after only 3 days of differentiation, the CTCF of MBP in rat oligodendrocytes cultured on 6.5 kPa PAHs functionalised with PDLMN was lower than that of OLs cultured on 6.5 kPa PAHs with PDL (Fig. 3B). The lower CTCF value found in the PDLMN condition may be explained by the marked decrease in cellular MBP area (Fig. 3D), but no changes on the intensity of the signal were observed (Fig. 3E). Concomitantly, the number of adherent cells per field after 3 days of differentiation was also significantly higher on 6.5 kPa PAHs functionalised with PDLMN than with PDL alone (*p* = 0.0351) (Fig. S3A), which could explain the observed decrease in cellular area due to higher cellular density and consequently lower CTCF of MBP. Concerning the MFI of MBP, it was slightly higher in presence of MN comparing with PDL alone after 3 days (Fig. 3E), showing the same trend as observed at day 5 of differentiation (Fig. 3G), although this increase was only statistically significant for the latter time point.

In summary, data indicate that the differentiation period of 5 days was the most appropriate time point for the differentiation of primary rat OPCs *in vitro* and that the combined presence of MN and compliant substrates enhanced the differentiation of the cells when compared with cells cultured on PDL alone, in contrast to what was observed on TCPs, where no significant differences were found between PDLMN *versus* PDL alone (Fig. 3C).

### Assessment of the maturation of OPCs into OLs

The maturation of oligodendrocytes cultured on the distinct platforms was assessed by analysing the expression of PLP (an oligodendrocyte maturation marker), using a similar approach as described above for the differentiation marker MBP. OPCs cultured for 3 days in differentiation conditions on 6.5 kPa PAHs in presence of MN displayed a higher CTCF value for PLP than cells cultured on PDL alone (*p* = 0.0051). This CTCF value was similarly high at day 5 of differentiation, but at this time point independent of the presence of MN (Fig. 4C, right panel). On the other hand, no effects of MN were observed on the maturation of OPCs when cultured on TCPs (Fig. 4C, left panel), suggesting that the presence of laminin-2 accelerated the maturation of oligodendrocytes only when cultured on compliant substrates (~6.5 kPa). Data also suggest that substrate compliance allows cells to eventually achieve a high level of maturation regardless of the presence of MN — as observed for cells after 5 days of differentiation cultured on 6.5 kPa PAHs (Fig. 4C, right panel). In fact the combined effect of laminin-2 and substrate compliance seems to be reflected instead by an early maturation of the cells, as observed by the increase of CTCF of PLP already at day 3 of differentiation on PAHs functionalised with PDLMN when compared to PDL alone (Fig. 4C, right panel).

After 3 days of differentiation, no significant differences were found regarding the area of PLP expression by PLP-positive oligodendrocytes cultured in the distinct conditions tested (Fig. 4D). However, cells differentiated for 5 days on 6.5 kPa PAHs displayed higher PLP area than those cultured on stiff TCPs (Fig. 4F), reinforcing the idea that substrate compliance favours OL maturation.

Regarding the influence of MN on the expression level of proteolipid protein, the MFI values obtained were higher in the presence than in the absence of MN after 3 days of differentiation on PAHs (*p* = 0.0165) (Fig. 4E, right panel), but not on TCPs (*p* = 0.7927) (Fig. 4E, left panel). No significant differences were found for cells on either substrate at a later stage (day 5) of differentiation (Fig. 4G). These results indicate a higher expression of PLP in response to laminin-2 only in cells cultured on compliant substrates at an earlier time point (3 days), but not on stiff TCPs.

Taken together, these results indicate that compliant substrates (6.5 kPa) promote the maturation of OPCs (mainly at the morphological level — Fig. 4F), and that the combined presence of MN and soft substrate leads to earlier expression of PLP (at day 3 of differentiation — Fig. 4E), suggesting earlier maturation of the cells (Fig. 4C).

### Compliant substrates favour the maturation of OLs, as evidenced by the formation of membranous- and myelin sheet-containing cells

**Figure 5:**
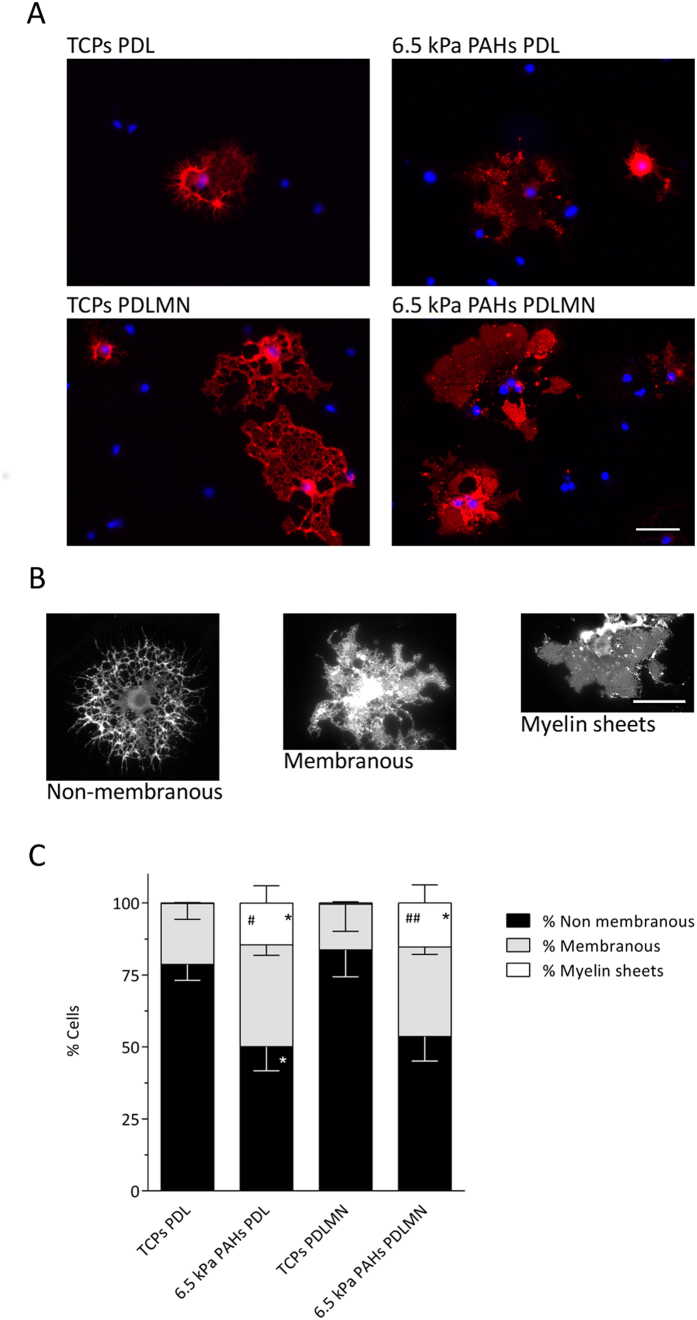
Morphological characterization of oligodendrocytes differentiated using distinct substrates and conditions. (**A**) Representative fluorescence microscopy images of primary rat oligodendrocytes cultured for 5 days in differentiation medium using distinct combinations of substrates (TCPs or 6.5 kPa PAHs) coated/functionalised with PDL or PDLMN, as indicated. Immunostaining was performed using an anti-MBP antibody (red) and DAPI for nuclear counterstaining (blue). Scale bars correspond to 50 μm. (**C**) Quantification of the percentage of cells bearing a non-membranous, membranous, or myelin sheet distribution of MBP, as depicted in the representative images in (**B**) — left, centre and right panels, respectively. The graph represents data from six independent experiments. Statistical analysis was performed by one-way ANOVA followed by Tukey’s multiple comparison test using the software GraphPad Prism 6. Statistically significant differences within cellular categories between substrates were represented (*, ^#^*p* < 0.05, ^##^*p* < 0.01, where *represent comparisons relative to TCPs PDL and ^#^comparisons relative to TCPs PDLMN).

Since it was not advisable to compare the CTCF and MFI of MBP and PLP directly between cells differentiated on distinct substrates because the materials (TCPs and PAHs) had potentially different optical properties, putatively interfering with the absolute values of MFI (panels C, E and G of Figs 3 and 4), a semi-quantitative approach was used in order to compare directly the maturation state of cells kept on the different platforms. For that end, MBP-positive oligodendrocytes were classified into three categories: (i) non-membranous, (ii) membranous and (iii) with myelin sheets (Fig. 5A,B). Myelin membrane sheets have been described to form only in the later stages of oligodendrocyte differentiation^39^, thus the formation of such myelin structures is restricted to highly mature OLs. The morphological classification of the MBP-positive cells showed that fully matured OLs displaying myelin sheets were essentially inexistent on TCPs after 5 days of differentiation, but were present on 6.5 kPa substrates (Fig. 5C). Moreover, there was also a significant increase in the percentage of membranous oligodendrocytes at the expense of non-membranous cells on PAHs when compared with TCPs (Fig. 5). These results were observed both in the presence and in the absence of merosin, in line with the PLP and MBP signal area results (Figs 3F and 4F) showing an overall higher morphological maturation state of the cells on PAHs than on TCPs.

Taken together, these results show that morphological maturation of OLs is highly dependent on the stiffness of the substrate, but less dependent on the presence of MN, which seems to play a more important role in terms of expression of differentiation (Fig. 3G) and maturation markers, which in case of PLP is also dependent on the combined presence of a compliant substrate (Fig. 4E).

### Fine-tuning of the extracellular matrix stiffness as a modulator of oligodendrocyte differentiation

**Figure 6:**
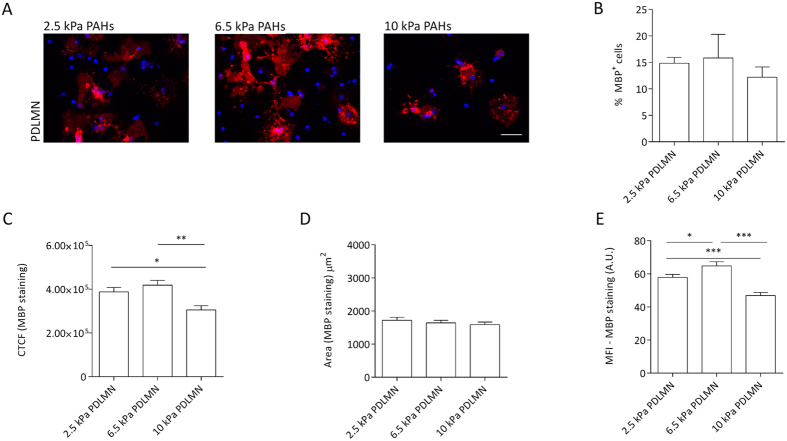
Modulation of oligodendrocyte differentiation and MBP levels by substrate stiffness within a narrow, brain-compliant range. (**A**) Representative fluorescence microscopy images of primary rat oligodendrocytes cultured for 5 days in differentiation medium using PAHs with distinct degrees of stiffness (2.5, 6.5 or 10 kPa) functionalised with^7^ PDL or PDLMN, as indicated, using an anti-MBP antibody (red) and DAPI for nuclear counterstaining (blue). Scale bars correspond to 50 μm. (**B**) Percentage of MBP-positive cells on the distinct substrates and (**C**) quantification of CTCF for MBP of oligodendrocytes cultured with differentiation medium for 5 days on 2.5, 6.5 and 10 kPa PAHs functionalised with PDL or PDLMN (as indicated). (**D**) Measurement of MBP signal area and (**E**) mean fluorescence intensity (MFI) of MBP signal (six fields per experiment from three independent experiments were analysed). Statistical analysis was performed by one-way ANOVA followed by Tukey’s multiple comparison test or Kruskal-Wallis test [in (**B**)] using the software GraphPad Prism 6. Statistically significant differences between substrate stiffness were represented using connectors (*p < 0.05, **p < 0.01).

**Figure 7:**
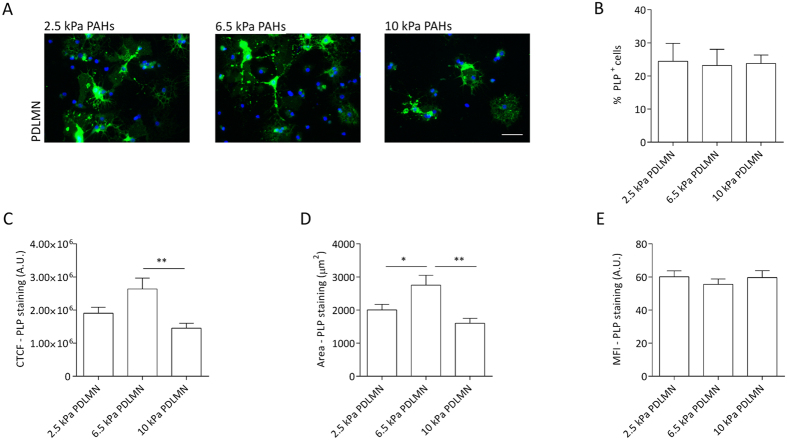
Modulation of oligodendrocyte maturation and PLP levels by substrate stiffness within a narrow, brain-compliant range. (**A**) Representative fluorescence microscopy images of primary rat oligodendrocytes cultured for 5 days in differentiation medium using PAHs with distinct degrees of stiffness (2.5, 6.5 or 10 kPa) functionalised with PDL +/− MN, as indicated, using an anti-PLP antibody (green) and DAPI for nuclear counterstaining (blue). Scale bars correspond to 50 μm. (**B**) Percentage of the PLP-positive cells on distinct substrates and (**C**) quantification of CTCF for PLP of primary oligodendrocytes cultured with differentiation medium for 5 days on 2.5, 6.5 and 10 kPa PAHs functionalised with PDL or PDLMN (as indicated). (**D**) Measurement of the PLP signal area and (**E**) mean fluorescence intensity (MFI) of the PLP signal (six fields from three independent experiments were analysed). Statistical analysis was performed by one-way ANOVA followed by Tukey’s multiple comparison or Kuskal-Wallis test [in (**B**)] using the software GraphPad Prism 6. Statistically significant differences between substrate stiffness were represented using connectors (*p < 0.05, **p < 0.01).

It was proposed that alterations of ECM stiffness could lead to anomalous myelination *in vivo*^13^. In order to evaluate more deeply the influence of substrate’s stiffness on the differentiation of OPCs *in vitro*, distinct cell-culture substrates with degrees of stiffness within the range of rigidity attributed to the brain — between 0.1 and 10 kPa^25^ — were tested. Namely, PAHs with Young’s moduli of 2.5, 6.5 and 10 kPa (gels number 3, 2 and 1, respectively — Table 1 and Fig. 1) functionalised with PDLMN were tested. After 5 days of differentiation, the percentage of MBP- and PLP-positive cells was similar between substrates (Figs 6A,B and 7A,B and S3B). However, differences were observed in terms of CTCF and MFI of MBP in cells cultured on the distinct substrates. The CTCF value was higher on 6.5 kPa PAHs than on 2.5 or 10 kPa, and the difference was statistically significant between 6.5 and 10 kPa PAHs (*p* = 0.0006) (Fig. 6C). The area of MBP signal was similar between conditions (Fig. 6D), nevertheless the MFI of MBP was statistically higher on 6.5 kPa PAHs than on the other substrates tested (Fig. 6E), indicating an increased level of MBP expression in OLs cultured on 6.5 kPa hydrogels. These results reinforced the idea that substrate rigidity influences the expression level of MBP in addition to the morphological maturation of the cells as already described in Figs 3F and 5.

Regarding PLP staining, the CTCF, area and MFI were also calculated. The CTCF for PLP was higher on 6.5 kPa PAHs than on other degrees of stiffness, being statistically significant between 6.5 and 10 kPa PAHs (*p* = 0.0011) (Fig. 7C), following the same trend as observed for MBP. The expression level of PLP seems to be similar between substrates, since the MFI did not change within the three conditions tested (Fig. 7E). However, the area of expression of PLP per cell was statistically higher on 6.5 kPa PAHs, supporting the idea that this particular stiffness promotes the differentiation and maturation of OPCs more than 2.5 or 10 kPa.

Overall, these results indicate that 6.5 kPa is the most appropriated stiffness within the tested range, suggesting that this could be close to the optimal rigidity to promote the differentiation and maturation of OPCs.

### Inhibition of non-muscle myosin-II promotes the elongation of processes of early-stage differentiation OPCs only when cultured on compliant substrates

**Figure 8:**
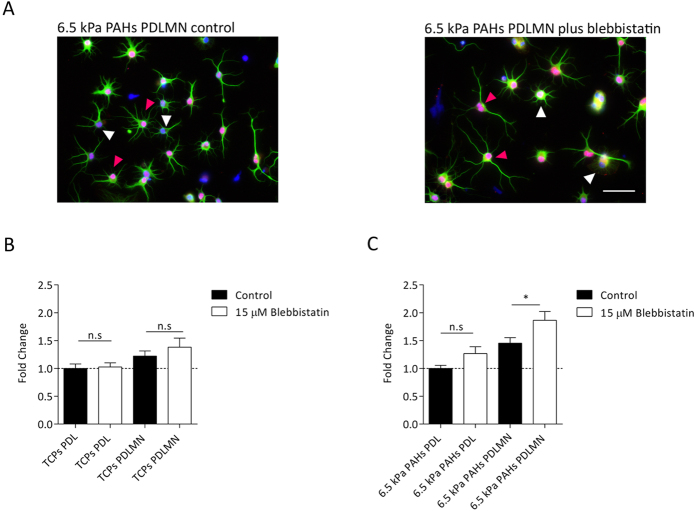
Effect of substrate stiffness, merosin and actomyosin contractility inhibition on morphological features of oligodendrocytes during early-stage differentiation. (**A**) Representative fluorescence microscopy images of OPCs cultured for 24 h in differentiation medium on 6.5 kPa PAHs functionalised with PDLMN in absence (left) or presence (right) of 15 μM of non-muscle myosin-II (NMM-II) inhibitor blebbistatin. Cells were immunostained for the oligodendrocyte lineage marker Olig2 (nuclear staining, in red) and for alpha-tubulin to stain the processes (green) and nuclei were counterstained with DAPI (blue). Scale bar corresponds to 50 μm. The area of oligodendrocyte processes of Olig2-positive cells — e.g.: cells highlighted by pink arrowheads in (**A**) — maintained on 6.5 kPa PAHs (**B**) or on TCPs (**C**) were normalised to the conditions PAHs PDL (**B**) or TCPs PDL (**C**). Data in graphs represent n = 30 cells obtained from three independent experiments. Statistical analysis was performed by *t*-test using the software GraphPad Prism 6. Statistical significance between conditions under comparison was represented using connectors (n.s.: non-significant, *p < 0.05).

The presence of the inhibitor of non-muscle myosin-II (NM-II) blebbistatin is known to induce relaxation of the cellular actin network and was already described to favour oligodendrocyte membrane extension during differentiation^24^,^40^. In such studies, cells were treated with blebbistatin throughout a period of 3 days in differentiating culture conditions, coincident with the expression of MBP, hence its effect was assessed only after differentiation being essentially achieved. In contrast, we aimed to understand the effect of actomyosin inhibition on early-stage differentiating oligodendrocytes. For that, primary rat OPCs were cultured in differentiation conditions on compliant substrates (6.5 kPa PAHs) or TCPs functionalised or coated (respectively) with PDL or PDLMN for 24 h, in presence or absence of blebbistatin. Cells were stained using antibodies against Olig2 and alpha-tubulin (to identify the oligodendrocyte-lineage specific cells and to stain the cellular processes, respectively) and the full area of the processes of oligodendrocytes was measured as a read-out of the process extension (Fig. 8A), as previously described by others^41^. In this experiment, only Olig2-positive cells were considered oligodendrocytes — Fig. 8A, pink arrowheads —, being the Olig2-negative cells considered as contaminating glia — Fig. 8A, white arrowheads.

It could be observed that blebbistatin promoted the extension of processes when OPCs were cultured on 6.5 kPa PAHs, notably only when comparing cells cultured on substrates functionalised with merosin, but not with PDL (Fig. 8B). Conversely, the effect of blebbistatin on OPCs cultured on TCPs was not statistically significant, when comparing the presence or absence of blebbistatin between PDL- or PDLMN-coated surfaces (Fig. 8C). It should be noted that for data represented in Fig. 8B,C the effect of MN in enhancing the extension of the processes of oligodendrocytes in absence of blebbistatin was also observed when comparing PDL- *versus* PDLMN-functionalised PAHs (*p* = 0.0003, two-tailed *t*-test), but not between PDL- and PDLMN-coated TCPs (*p* = 0.068, two-tailed *t*-test).

These results indicate that the combined effect of blebbistatin, compliant substrates and presence of merosin promotes the early (24 h) differentiation of OPCs.

## Discussion

Oligodendrocyte progenitor cells (primary rat OPCs and CG-4 cells) responded to mechanical and biochemical cues provided by brain-compliant substrates functionalised with ECM proteins, changing morphological complexity and expression of differentiation and maturation markers (MBP and PLP, respectively).

Non-differentiated CG-4 cells exhibited distinct morphological features when cultured in proliferation medium (under non-differentiating conditions) in presence of FN or MN, exhibiting a bipolar morphology characteristic of oligodendrocyte progenitors or a branched morphology typical of differentiated cells, respectively. This effect was only statistically significant when cells were cultured on brain-compliant substrates — 6.5 kPa, as assessed by rheometry (Fig. 1 and Table 1) —, but not when kept on stiff TCPs (Fig. 2B). These results indicate that oligodendrocyte progenitor cells become particularly responsive when cultured on compliant substrates to the well-established effect of fibronectin of maintaining the progenitor state of oligodendrocytes and the effect of laminin-2/merosin (MN) of inducing the differentiation of the cells^15^,^16^,^17^,^18^,^19^,^20^, even in presence of non-differentiating medium (as reported here). Such morphological changes may be related with changes in the contractility of actomyosin of OLs in response to soft substrates, in agreement with what has been described in the literature^24^,^41^,^42^, but our results allow us to speculate that, additionally, the signalling pathways activated by FN and MN known to favour the maintenance of the precursor state or the differentiation of oligodendrocytes (respectively) are enhanced by brain-compliant substrates.

Further experiments using primary oligodendrocytes reinforced this idea, since the maturation of cells differentiated in presence of MN was higher when comparing with cells cultured on PDL-coated substrates, but only when kept on compliant PAHs. A significant increase of CTCF values for MBP and PLP (which take into account the level of expression of MBP or PLP and the morphological maturation — by assessing the MFI value and area of signal, respectively) was observed when cells were differentiated on PAHs, but not when maintained on stiff TCPs substrates (Figs 3C and 4C).

At day 5 of differentiation, OLs maintained in presence of merosin showed increased MBP expression (in terms of MFI) when compared with cells kept on PDL alone. This effect was observed both on TCPs — in agreement with previous reports^16^,^18^ — and on PAHs (Fig. 3G). However, the combined effect of MN and substrate compliance led to an increased extent of MBP expression (in terms of area of expression within the cell), denoting higher maturity of OLs when compared with cells kept on stiff substrates in presence or absence of MN (Figs 3F and S2), reinforcing the idea that both stimuli combined contribute to an enhanced differentiation and maturation of OLs.

Additionally, higher expression of PLP induced by MN was observed after only 3 days in differentiation conditions, but only for oligodendrocytes maintained on brain-compliant substrates (Fig. 4E), again suggesting that enhanced maturation of OLs occurs in conditions combining the presence of MN and soft substrates. Substrate compliance (6.5 kPa PAHs) also led to an increased extent of PLP expression after 5 days of differentiation (concerning the area of expression within the cell — Fig. 4F), indicating higher maturation of the cells in comparison with those on stiff substrates.

By categorizing differentiated OLs (only considering MBP-positive cells) based on the presence, complexity or absence of myelin sheets (Fig. 5), it was confirmed that morphological maturation of oligodendrocytes is highly influenced by mechanical properties of the substrate. In our culture conditions, OLs displaying well-formed myelin sheets only became present when differentiated on 6.5 kPa PAHs, but not on stiff TCPs. Moreover, there was also an increase in the percentage of less developed but still clearly membranous cells, and to be noted, the proportion of the two more mature cell populations increased at the expense of the most immature, non-membranous one (Fig. 5C).

Overall, our results indicate that protein levels of MBP and PLP (assessed by measuring the MFI of immunocytochemistry images) were enhanced by the presence of MN — and only on compliant substrates in case of PLP —, whereas the morphological maturation of the cells seems to be more dependent on substrate stiffness than on the ECM proteins used to functionalise the substrates tested (Figs 3, 4, 5), although in case of MBP area, the presence of merosin also seems to play a role (Fig. 3F). Our results are in agreement with results from others, showing an increase of cellular area and morphological complexity of OLs differentiated on soft substrates^23^,^24^, although such studies presented some limitations (as detailed below).

On previous studies (indicated above), the levels of expression of differentiation/maturation markers like MBP and PLP were not addressed. Additionally, to our knowledge, the only type of functionalization reported so far in the literature in an oligodendroglial context was performed using poly-D-lysine (PDL), which allows cell adhesion, but without direct engagement of integrins. Since it is well known that merosin/integrin interactions promote myelin membrane formation in oligodendrocytes^16^ and play a key role during differentiation of OPCs into OLs, our study was particularly focused on testing the combined effect of ECM proteins in a brain-compliant context. Based on our results, we propose that the combination of appropriate stiffness with the extracellular matrix protein merosin has a cumulative positive effect during oligodendrocyte differentiation, possibly by potentiating the activation of integrins and downstream players relevant for the differentiation of OLs^20^,^43^.

Another limitation found in the literature was related with the range of stiffness degrees tested, since the intervals reported so far were either limited^24^ or presented very wide intervals, not exploring properly the rigidity range between 1 and 10 kPa^23^, which is especially relevant in mechanotransduction studies concerning CNS cells. Our initial experiments were also performed by comparing two substrates presenting values of stiffness several orders of magnitude apart — ~1 GPa TCPs and 6.5 kPa PAHs (Figs 2, 3, 4, 5). Additionally, TCPs and PAHs also differed from each other in terms of other properties that could putatively influence cell behaviour, such as different surface chemistry and adhesiveness for coated proteins. For these reasons, distinct formulations of PAHs within the stiffness range of 1 and 10 kPa (2.5, 6.5 and 10 kPa) were further tested, in order to fine-tune the optimal conditions to promote the differentiation of OPCs and to rule out that the cell behaviour observed in response to the distinct substrates could be caused by unaccounted factors. Our results showed that 6.5 kPa seems to be the optimal stiffness to promote the differentiation and maturation of oligodendrocytes (Figs 6 and 7). The CTCF values for MBP and PLP showed a similar trend between conditions and were statistically higher on 6.5 kPa than on 10 kPa PAHs. Additionally, the increase of the CTCF value of MBP was mainly influenced by a higher level of expression of this marker in terms of MFI (Fig. 6D,E), whereas the CTCF value of PLP was mostly dependent on the area of expression within the cell (Fig. 7D,E), suggesting that distinct mechanisms might direct the behaviour of these proteins.

Taken together, our results are in agreement with the proposed idea that myelination/remyelination processes could be affected by tissue stiffness, i.e. extracellular tissue environments which are softer or stiffer than physiologically normal could lead to anomalous myelination/remyelination, contributing to demyelinating chronic diseases, such as multiple sclerosis^13^. Furthermore, our results suggest that the optimal stiffness to promote the differentiation/maturation of oligodendrocytes is around 6.5 kPa, well within the physiological range of stiffness described in the literature for the brain^25^.

It is known that mechanical stimuli are transmitted through modulation of the cytoskeleton and activation or inhibition of actomyosin contractility (reviewed in Clark, K. *et al*.)^44^. The presence of blebbistatin (inhibitor of actomyosin contractility) in oligodendroglial cultures leads to an enhancement of OL maturation, contributing to increased cellular branching, process length, area, and percentage of MBP^ + ^cells^24^,^40^,^41^. However, all previous studies assessed the long-term effect of contractility inhibition of actomyosin, essentially assessing cellular phenotype only at the end of the differentiation process. Thus, we sought to study the combined effect of blebbistatin and substrate rigidity in early-stage differentiation of OPCs (i.e., 24 h of culture in differentiation conditions) on TCPs and compliant substrates (6.5 kPa PAHs), which had not been reported yet. Our studies showed that the inhibition of non-muscle myosin-II (NMM-II) using blebbistatin during the first 24 h of differentiation had a positive impact in the early acquisition of differentiation morphological features of oligodendrocytes, however these effects were only observed when cells were cultured on a compliant (6.5 kPa), but not on a stiff substrate (~1 GPa TCPs) — Fig. 8. This finding provides valuable information concerning the effect of a transient (as opposed to long term) use of this drug, with implications in terms of future translational applications and the development of medicinal drugs targeting myosin.

Finally, to fully understand the mechanisms involved in the conversion of mechanical cues into biochemical signalling, the role of the cytoskeleton on such pathways and how this information can be used to facilitate the differentiation of oligodendrocytes, and eventually contribute to myelination/remyelination processes, will require further mechanistic studies.

## Conclusion

The use of 6.5 kPa substrates (as assessed by rheometry) combined with the ECM protein laminin-2/merosin could significantly improve the differentiation of OPCs into OLs. Moreover, OLs cultured on brain compliant substrates presented a more mature morphology and increased expression of MBP and PLP. These findings indicate that physical cues in combination with appropriate biochemical stimuli provided by cell culture substrates mimicking the extracellular matrix play a major role in the differentiation of oligodendrocytes, which pose important implications for future cell- and bioengineering-based therapeutic approaches.

## Materials and Methods

### Preparation of polyacrylamide hydrogels (PAHs)

Polyacrylamide hydrogels (PAHs) were prepared using a protocol based on Cretu, A. *et al*.^45^ with modifications. Hydrogels were assembled on top of reactive glass coverslips, allowing the establishment of covalent bonds between the hydrogel and the glass^46^. Coverslips were activated by treating with 3-(trimethoxysilyl)propyl methacrylate diluted 1:200 (v/v) in absolute ethanol, which, immediately before use, was supplemented with 3% (v/v) of diluted acetic acid (diluted 1:10 in water). This solution was placed on top of previously cleaned and sterile coverslips and allowed to react for 3 minutes, and then rinsed with ethanol and air-dried inside a tissue culture laminar flow cabinet.

The solutions to produce the PAHs were prepared by mixing (in a sterile conical tube) sterile deionised water, 40% acrylamide (Bio-Rad) and 2% bis-acrylamide (AppliChem) at the desired final percentages (Table 1), and then TEMED (0.01% v/v). The pH of the polyacrylamide solution was adjusted to 7.5 by adding HCl (2 N) and then the solution was degassed for 30 minutes^47^, using a vacuum system (Vacusafe, Integra Biosciences). Then, N-acrylosuccinimide ester — NHS (Santa Cruz Biotechnology) was added to the polyacrylamide solution to allow the subsequent functionalization of the hydrogels with proteins or other polypeptides. The NHS solution was prepared in toluene (20 mg/mL). After degassing, 0.03% (v/v) APS and 22% (v/v) of the previously prepared NHS solution was slowly added to the acrylamide-containing solution and then mixed carefully by inverting the conical tube, avoiding the formation of air bubbles.

The PAHs were polymerised using an electrophoresis gel casting system with 1 mm spacers (Mini-protean III, Bio-Rad), since it was described that hydrogels with thickness larger than 100 μm are sufficiently thick to prevent cells from sensing a stiff glass coverslip that the gel might be attached to^48^ (as it is the case for the PAHs described here). The spacer-containing glass and the outer glass were chloro-silanated with 5% (v/v) dichlorodimethylsilane — DCDMS in toluene for five minutes^49^ to avoid adherence of the hydrogels to the surfaces, and then dried with a wipe. The reactive coverslips were placed on top of the treated spacer-containing glass (the coverslips adhered to the glass surface by using small droplets of water between both glass surfaces). Next, the system was mounted and the PAH solution was pipetted slowly between the activated coverslips (adherent to the spacer-containing glass) and the outer glass and allowed to polymerize for 30 minutes at room temperature (RT). Then, hydrogels covalently linked to the coverslips were removed from the system and washed three times with sterile 1× DPBS pH 7.4, five minutes each with gentle agitation. The hydrogels were placed on tissue culture polystyrene plates — TCPs (Corning-Costar) and sterilised under UV light for 30 minutes inside a class-II air-flow biosafety cabinet. All procedures to prepare PAHs were carried out using an air-flow cabinet except the treatment of the spacer-containing glass and the outer glass with DCDMS that was performed using a chemical hood.

PAHs were functionalised by adding the desired protein/polypeptide solution diluted in sterile 1× DPBS pH 7.4 at a concentration of 25 μg/mL on top of the hydrogel, followed by overnight (ON) incubation at 4 °C. In this study, the following proteins/polypeptides were used: fibronectin — FN (Roche), merosin — MN (Merck-Millipore) and Poly-D-lysine — PDL (Sigma-Aldrich). Combination of PDL and MN (further on referred to as PDLMN) was prepared 1:1 using each solution at a final concentration of 25 μg/mL. On the following day, the hydrogels were rinsed with sterile DPBS and blocked with inactivated BSA (1 mg/mL) in Dulbecco’s Modified Eagle’s Medium (DMEM) low glucose — 1000 mg/L (Life Technologies). BSA was previously inactivated at 68 °C for 30 minutes. Finally, the PAHs were washed with DPBS and equilibrated in DMEM low glucose for 4 h before cell seeding. As control, glass coverslips or TCPs were coated with the same proteins at the same concentration (glass coverslips were incubated for 4 h at 37 °C and TCPs ON at 4 °C).

### Characterization of polyacrylamide hydrogels (PAHs)

#### Rheological assessment of PAHs by rheometry

The rheological characterization of hydrogels was determined by small-strain oscillatory shear tests using a Kinexus Pro rheometer and rSpace software (Malvern) fitted with a parallel plate geometry (stainless steel wrinkled plate, 4 cm diameter). The hydrogels were prepared and polymerised following a protocol similar to the one described above to produce hydrogels for cell culture, except that the gels were not linked to coverslips or functionalised with protein. After zeroing the rheometer, each gel was loaded and trimmed on the bottom plate. Then, the gap (distance between the top and bottom plates) was defined as 1 mm and fine-tuned to a distance at which the gel was subjected to a normal force of 0.1 N. Frequency sweeps were performed from 10 to 0.1 Hz (three reads per decade) with a deformation of 2 millistrain at 37 °C. The tensile elastic modulus (*E* — Young’s modulus) was calculated from the measured viscoelastic shear modulus using the formula *E* = 2*G*′(1 + *ν*), where *G*′ is the shear storage modulus measured at 1 Hz and *ν* is Poisson’s ratio, assumed to be 0.5 for materials that do not vary its volume upon stretch^22^,^25^.

#### Rheological assessment of PAHs by atomic force microscopy (AFM)

Force-distance spectroscopy-based nanomechanical analysis was performed using a Nanosurf Flex-ANA system (Nanosurf AG, Liestal, Switzerland) equipped with commercial soft contact mode cantilevers (qp-SCONT, Nanosensors, Neuchâtel, Switzerland; nominal spring constant 0.01 N/m). The cantilever spring constant was calibrated prior to each experiment using the Sader method^50^. For each measurement, three to four different polyacrylamide gels bonded to a glass cover slip were fixed inside a single plastic Petri dish using fast curing two-component epoxy glue. The gels were then submerged in freshly prepared PBS buffer. Force-distance maps were recorded automatically on pre-defined location on the gels. At each location, a 75 × 75 μm^2^ force map consisting of 1024 evenly distributed force curves was recorded. Thereto, the cantilever was approached towards the sample with 2 μm/s until the cantilever tip indents the sample and a contact force of 500 pN was reached. Then, the cantilever was retracted from the surface at the same velocity. During such an approach-retract cycle, the cantilever deflection was recorded along with the z-piezo position.

The Nanosurf Flex-ANA analysis software (Nanosurf AG, Liestal, Switzerland) was used to analyse force distance curves applying the conical indenter Sneddon model^51^ with a sample Poisson ratio of 0.5. The Sneddon model has been shown to be applicable to the analysis of force-distance curves acquired on soft biological material and hydrogels^52^,^53^,^54^. For each formulation, the elastic moduli measured on 9 to 16 locations on at least two independent preparations were pooled. From the pooled data, a histogram was compiled and then fitted using a Gaussian distribution.

#### Determination of the swelling ratio of PAHs

To measure the swelling ratio (SR), polyacrylamide hydrogels were produced as described above for the rheological assessment by rheometry (not linked to coverslips or functionalised with protein). PAHs were weighted after three washes with PBS (initial weight) and then weighted again after immersing in PBS for 24 h, followed by quickly drying the excess liquid with filter paper (final weight). SR was calculated by dividing the final weight by the initial weight.

### Cell culture

#### Culture of CG-4 cells

The CG-4 cell line (kindly provided by Dr. Adil J. Nazarali, College of Pharmacy and Nutrition, university of Saskatchewan, Saskatoon, Canada) was maintained as reported^31^. Briefly, cells were harvested by washing three times with Puck´s solution^55^, followed by dissociation and detachment using Trypsin (500 μg/mL)-EDTA (200 μg/mL) solution (Life Technologies). Trypsin was inactivated by adding 10× the volume of recovery medium [DMEM high glucose — 4500 mg/L (Hyclone), with 5% (v/v) FBS (Life Technologies) sodium pyruvate (2 mM), human insulin (5 μg/mL) — both from Sigma-Aldrich —, Penicillin (10 U/mL), Streptomycin (10 μg/mL) and Amphotericin B (2.5 μg/mL) — all from Life Technologies] and cells were centrifuged at 200 × *g* for 5 minutes at RT, re-suspended, counted and seeded on tissue culture polystyrene plates (TCPs) previously coated with PDL (100 μg/mL, ON at 37 °C) at a density of 2,500 cells/cm^2^ in recovery medium. CG-4 cells were allowed to adhere for 30 minutes inside a CO_2_ incubator at 37 °C, 5% CO_2_/95% air and 95% humidity. After cell attachment, the recovery medium was replaced by CG-4 proliferation medium composed by DMEM high glucose supplemented with apo-transferrin (50 μg/mL), biotin (9.8 ng/mL), sodium selenite (40 ng/mL) — all supplements from Sigma–Aldrich —, 30% of B104 cell line – conditioned medium, Penicillin (10 U/mL), Streptomycin (10 μg/mL) and Amphotericin B (2.5 μg/mL). Cells were maintained in the CO_2_ incubator at 37 °C and the medium was changed every other day^56^. CG-4 cells were cultured on functionalised PAHs with FN or PDLMN and, as control, on TCPs coated with the same proteins at a density of 6,400 cells/cm^2^ for 2 days.

#### Culture of B104 cells and preparation of conditioned medium

B104 neuroblastoma cells (kindly provided by Dr. Adil J. Nazarali) were maintained in B104 proliferation medium [DMEM/F12 (Life Technologies) supplemented with 10% (v/v) FBS, Penicillin (10 U/mL), Streptomycin (10 μg/mL) and Amphotericin B (2.5 μg/mL)], at 37 °C, 5% CO_2_/95% air and 95% humidity. To obtain conditioned medium, cells were previously seeded at a density of 15,000 cells/cm^2^ in proliferation medium for 24 h. Then, cells were washed three times with Puck’s solution and the medium was replaced by defined medium, composed by DMEM/F12 supplemented with holo-transferrin (10 μg/mL), sodium selenite (5 ng/mL), putrescine (16 μg/mL), progesterone (6.3 ng/mL) — all from Sigma–Aldrich — and Penicillin (10 U/mL), Streptomycin (10 μg/mL) and Amphotericin B (2.5 μg/mL). Three days later, the conditioned medium was collected and was added PMSF (1 μg/mL). Next, the medium was centrifuged at 1,000 × *g* at 4 °C for 10 minutes and the supernatant was filtered (0.22 μm filter) and stored at −20 °C^56^.

#### Isolation of primary oligodendrocyte progenitor cells (OPCs)

The experimental methods involving animals were approved by the IBMC Animal Ethics Committee and licensed by the Portuguese Veterinary Office (DGAV). All animal procedures were carried out in strict accordance with the European Directive 2010/63/EU and DGAV General guidelines. Primary rat OPCs were isolated from cerebral cortices from P0-P2 rat pups, using a procedure based on McCarthy, K.D. & de Vellis, J.^57^ and Milner, R. & ffrench-Constant, C.^58^ with minor modifications. First, the skull was removed, the cortices were isolated, and the meningeal membrane was removed with forceps (during the procedure the tissue was maintained on ice). Then, each cortex was transferred to a conical tube (about 10 cortices per tube) containing 1 mL DMEM high glucose (4500 mg/L, from Life Technologies) containing Penicillin (10 U/mL) and Streptomycin (10 μg/mL) and was mechanically dissociated by pipetting several times, and then the suspension went through a 25 G needle using a sterile syringe. Afterwards, the tissue was enzymatically dissociated by adding Trypsin (2.5 mg/mL)-EDTA (380 μg/mL) and DNase (50 μg/mL) and incubated at 37 °C for 15 minutes. Trypsin was inactivated by adding DMEM high glucose supplemented with 10% (v/v) FBS, Penicillin (10 U/mL) and Streptomycin (10 μg/mL). Then, the suspension was centrifuged at 500 × *g* for 10 minutes and the pellet was re-suspended in DMEM supplemented with serum and antibiotics (as described for trypsin inactivation), and the cell suspension went through a cell strainer (mesh size of 100 μm) to a new tube. Cells were cultured on tissue culture T75-flasks (Corning-Costar) previously coated with PDL (10 μg/mL), and were maintained for at least 10 days with DMEM high glucose supplemented with serum and antibiotics. The medium was changed every 2 days. OPCs were collected by shaking the flasks at 270 rotations per minute (rpm) at 37 °C overnight under hypoxia conditions. OPCs were purified from the suspension by plating cells on bacteriological plastic dishes for at least 1 h at 37 °C inside the CO_2_ incubator (so that contaminant cells became adherent) and then the OPCs (non-adherent cells) were centrifuged at 500 × *g* for 15 minutes. Next, the cells were re-suspended, counted and seeded on tissue culture plates previously coated with PDL (10 μg/mL) at a density of 16,000 cells/cm^2^ and maintained in proliferation medium (PM), based on Sato’s medium^59^ with slight modifications [DMEM high glucose (Hyclone), glutamine (2 mM, from Life Technologies), apo-transferrin (100 μg/mL), BSA (100 μg/mL), sodium selenite (40 ng/mL), human insulin (5 μg/mL), progesterone (60 ng/mL), putrescine (16 μg/mL), triiodo-L-thyronine/T3 (30 ng/mL), thyroxin/T4 (40 ng/mL, all from Sigma–Aldrich), bFGF (10 ng/mL) and PDGF-AA (10 ng/mL, both from Peprotech) and Penicillin (10 U/mL), Streptomycin (10 μg/mL) and Amphotericin B (2.5 μg/mL)].

#### Culture of primary OPCs

Primary rat OPCs were washed three times with Puck’s solution and detached using Trypsin (500 μg/mL)-EDTA (200 μg/mL) solution for 5 minutes at 37 °C. Trypsin was inactivated by adding 10x the volume of CG-4 recovery medium and OPCs were centrifuged at 300 × *g* at RT for 15 minutes and re-suspended in PM and cultured at a density of 17,000 cells/cm^2^ on PAHs or TCPs (both substrates were prepared as described above for CG-4 cells). OPCs were allowed to adhere for 1 hour, after which the medium was switched to proliferation or differentiation medium (DM). DM was similar to Sato’s medium without bFGF or PDGF-AA plus 0.5% (v/v) FBS. OPCs were maintained for 2 days with PM and 3 or 5 days with DM.

In experiments where the non-muscle myosin-II was inhibited, OPCs were cultured in the presence or absence of racemic ( ± )-blebbistatin (15 μM) in differentiation medium for 24 h.

### Immunocytochemistry and agglutinin staining

To stain CG-4 cells and OPCs, the medium was removed and cells were washed once with Puck’s solution and then added the fixation solution: 4% (w/v) paraformaldehyde (PFA) in PBS for 15 minutes at RT. Fixed cells were then washed with PBS (for immunocytochemistry) or with HBSS (for agglutinin staining). For agglutinin staining, fixed cells were incubated with FITC-labelled wheat germ agglutinin (5 μg/mL) for 1 h at RT. Immunocytochemistry was performed as detailed in Leite, C. *et al*.^60^ using the antibodies (and respective dilutions) summarised on Supplementary Table 1. When using anti-PLP antibody, cells were treated with glycine (20 mM) for 5 minutes at RT before permeabilization. Fluorescence microscopy was performed using a Zeiss Axiovert 200 M microscope using AxioVision Release 4.8 software (Zeiss) for image acquisition. Exposure time was the same for each marker analysed and for each independent experiment.

### Image analysis and quantification of fluorescence microscopy images

#### Fractal dimension analysis

Fractal dimension (D) analysis has been applied to cell biology^32^,^33^ to evaluate morphological complexity of cells, rendering a numerical value close to 1 for cells with low morphological complexity (essentially bipolar cells) and near 2 for those with high complexity (highly branched cells or with a bi-dimensional planar structure). D was calculated using the Image J software v1.49 (NIH). In detail, each fluorescence microscopy image was converted to 8-bit and then using the ‘Crop’ tool (‘Image’-‘Crop’), one cell was cropped and the ‘Threshold’ (‘Image’-‘Adjust’-‘Threshold’) was adjusted in order to select the whole cell. Finally, the cell was outlined using the ‘Outline’ tool (‘Process’-‘Binary’-‘Outline’) and in ‘Analyse’, the ‘Tools’ command was chosen. Next, the ‘Fractal box count’ was selected and the corresponding D value was obtained. This procedure was performed for at least four cells per condition for each independent experiment (*n* = 3).

#### Quantification and classification of MBP- and PLP-positive cells

To calculate the percentage of MBP- and PLP-positive oligodendrocytes, cells were counted from at least six random fields per condition for each independent experiment. To determine the total number of cells, nuclei (stained with DAPI) were counted using Image J tool ‘Analyse particles’ after setting the matching interval of size for individual nuclei, and MBP- or PLP-positive cells were counted manually, in order to determine the percentage of cells positive for each marker.

The morphology of MBP-positive cells was classified according to branching and membrane complexity: (1) non-membranous, for cells expressing MBP with either a complex or simple morphology; (2) membranous, for complex cells containing at least one membranous region; and (3) myelin sheets, for cells displaying a fully planar MBP membrane.

The quantification of MBP and PLP from immunofluorescence images (using Image J) was performed as described in Leite, C. *et al*.^60^ with modifications, by analysing six fields belonging to each condition from at least three independent experiments. Briefly, random fields were selected and converted to 8-bit TIFF images, and then all MBP- or PLP-positive cells in the fields were individually selected by outlining (using the ‘Freehand selections’ tool) and creating regions of interest (ROI) corresponding to each individual cell (‘Analyse’-‘Tools’-‘ROI Manager’-‘Add’, or ‘Ctrl + t’). Next, for each ROI, the signal was determined by adjusting the threshold (using the ‘Threshold’ tool) in order to select the whole cell. The mean fluorescence intensity (MFI), area and integrated density of the signal were measured (‘Analyse’-‘Set Measurements’-check ‘limit to Threshold’, ‘Mean grey value’, ‘Area’ and ‘Integrated density’, followed by ‘Measure’ in the ROI Manager window). The same was performed to determine the background, except that the threshold was defined by inverting the selection.

For MFI analysis, the MFI of the signal was subtracted by the MFI of the background. The corrected total cell fluorescence (CTCF) was calculated as follows: CTCF = Integrated density – (MFI of the background x Area of the signal) — Fig. S4. Corrected MFI, area and CTCF were averaged and subjected to statistical analysis.

#### Measurement of process area of early differentiating oligodendrocytes

The area of processes of early differentiating oligodendrocytes was measured (using Image J) on immunofluorescence images obtained after α-tubulin staining of OPCs cultured in differentiation medium for 24 h. Only OPCs (Olig2-positive cells) with at least three main processes (considered as cells initiating the differentiation process) were analysed. Cells were selected using the ‘Threshold’ tool and the area was measured, then the cell body was outlined and its area was also measured. Next, the area of processes was calculated by subtracting the cell body area to the total cellular area. The results from ten cells per independent experiment (n = 3) were averaged and subjected to statistical analysis.

### Protein extraction and quantification, SDS-PAGE, and immunoblotting

To obtain protein extracts from primary rat oligodendrocytes in culture, cells were scraped with Laemmli buffer [Tris-HCl (120 mM) pH 6.8, SDS (4% w/v) and glycerol (20% v/v)]. The extract from each experimental condition was collected to a microtube and heated at 95 °C for 5 minutes. Afterwards, the sample was spun down and passed through a 25 G needle for ten times. Before loading the extracts on polyacrylamide electrophoresis gels for separation, each sample was added DTT (0.1 M final concentration). Protein samples were separated by SDS-PAGE as previously described^60^. For immunoblotting, membranes were blocked with blocking solution, consisting of PBS-T [PBS with Tween 20 (0.1% v/v)] with non-fat dried milk (5 g/100 ml) for 1 h at RT. Incubation with anti-MBP and anti-tubulin antibodies were performed with gentle agitation, overnight at 4 °C, followed by 1 h at RT. Membranes were washed with PBS-T and then incubated with the respective secondary antibody diluted in blocking solution (1 h at RT) and washed with PBS-T. The antibodies used and the corresponding dilutions are described in the Supplementary Table 1. An ECF (enhanced chemifluorescence) kit (GE Healthcare) was used for detection of the alkaline phosphatase-conjugated antibodies, according to the manufacturer’s instructions. Alkaline phosphatase activity (using ECF) was visualised on a Molecular Imager FX Pro Plus system (BioRad) using the software Quantity One (BioRad). In order to quantify the total protein in each entire lane, the membranes were stained using a SERVA purple kit (SERVA electrophoresis) according to the manufacturer’s instructions and images were acquired using a Molecular Imager FX Pro Plus system using the software Quantity One. The integrated density of the western blot and total protein bands was measured using Quantity One software.

### Statistical analysis

Statistical analysis was performed by using two-tailed Student’s *t-test,* one-way ANOVA followed by Tukey’s test, or two-way ANOVA followed by Bonferroni post-test, as indicated in the legends of figures. When the data did not pass Shapiro-Wilk normality test and sample size was lower than 30, the non-parametric Kruskal–Wallis one-way ANOVA followed by Dunn’s multiple comparison test was used. Analysis was done using the software GraphPad Prism 6. Values represent mean ± SEM or mean ± SD, as indicated, of at least three independent experiments (*^, #^*p* < 0.05; **^, ##^*p* < 0.01 and ***p < 0.001 for statistically significant differences).

## Additional Information

**How to cite this article**: Lourenço, T. *et al*. Modulation of oligodendrocyte differentiation and maturation by combined biochemical and mechanical cues. *Sci. Rep.* **6**, 21563; doi: 10.1038/srep21563 (2016).

## Supplementary Material

Supplementary Information

## Figures and Tables

**Table 1:** Formulation (in percentage of acrylamide — AC — and bis-acrylamide — BAC), swelling ratio and Young’s modulus measured by rheometry of distinct polyacrylamide hydrogels (numbers 1–6).

Gel number	% AC/% BAC	Swelling ratio Mean ± SD	*E* – Young’s modulus (Pa) Mean ± SD
1	12.5%/0.37%	1.06 ± 0.024	9720 ± 1352
2	10%/0.3%	1.05 ± 0.034	6629 ± 2691
3	5%/0.2%	1.06 ± 0.041	2442 ± 858
4	4%/0.2%	1.09 ± 0.036	2032 ± 738
5	3%/0.2%	1.14 ± 0.048	952 ± 320
6	3%/0.05%	1.40 ± 0.140	362 ± 65
