# Supplementary material for: Modulation of oligodendrocyte differentiation and maturation by combined biochemical and mechanical cues
Source: Sci Rep. 2016 Feb 16;6:21563. doi: 10.1038/srep21563 (PMC4754901; doi:10.1038/srep21563)
Supplement: Supplementary Information [file srep21563-s1.pdf]

Title:

Modulation of oligodendrocyte differentiation and maturation by combined biochemical and mechanical cues

Authors:

Tânia Lourenço[1,2], Joana Paes de Faria[3,4], Christian A. Bippes[5], João Maia[6], José A. Lopes-da-Silva[7], João B. Relvas[3,4], Mário Grãos[1,2*]

Affiliations:

[1] Biocant, Technology Transfer Association, Cantanhede, Portugal

[2] Centre for Neuroscience and Cell Biology (CNC), University of Coimbra, Coimbra, Portugal

[3] IBMC – Instituto de Biologia Molecular e Celular, Universidade do Porto, Porto, Portugal

[4] i3S - Instituto de Investigação e Inovação em Saúde, Universidade do Porto, Portugal

[5] Nanosurf AG, Liestal, Switzerland

[6] Chemical Engineering Department, Faculty of Science and Technology, University of Coimbra, Coimbra, Portugal

[7] QOPNA, Chemistry Department, University of Aveiro, Aveiro, Portugal

* Corresponding author. Email: mgraos@biocant.pt

Supplementary information

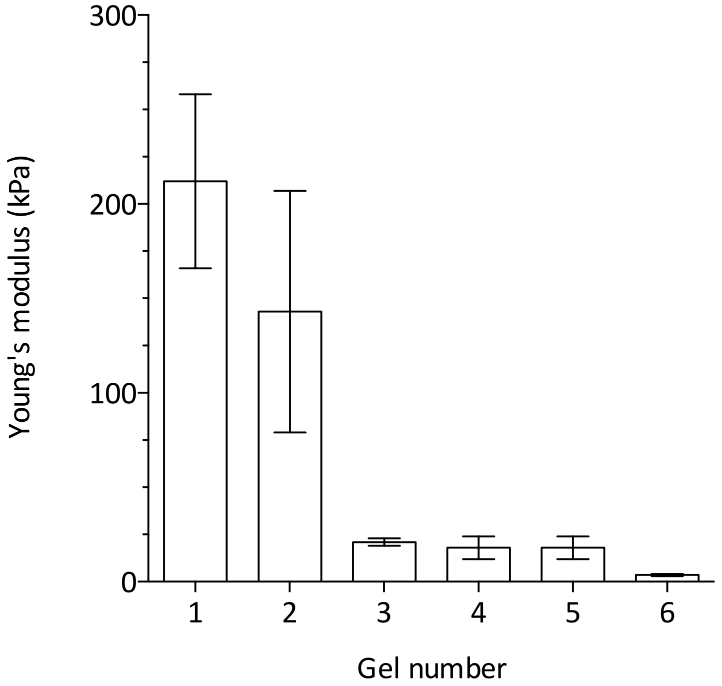

Figure S1 - Young's moduli of polyacrylamide gels determined by AFM. The Young's moduli were measured using force-distance curve-based AFM nanoindentation. For each gel formulation measurements were performed on at least two independent gel preparations. Data represent mean ± SD of the Young's modulus of each gel formulation.

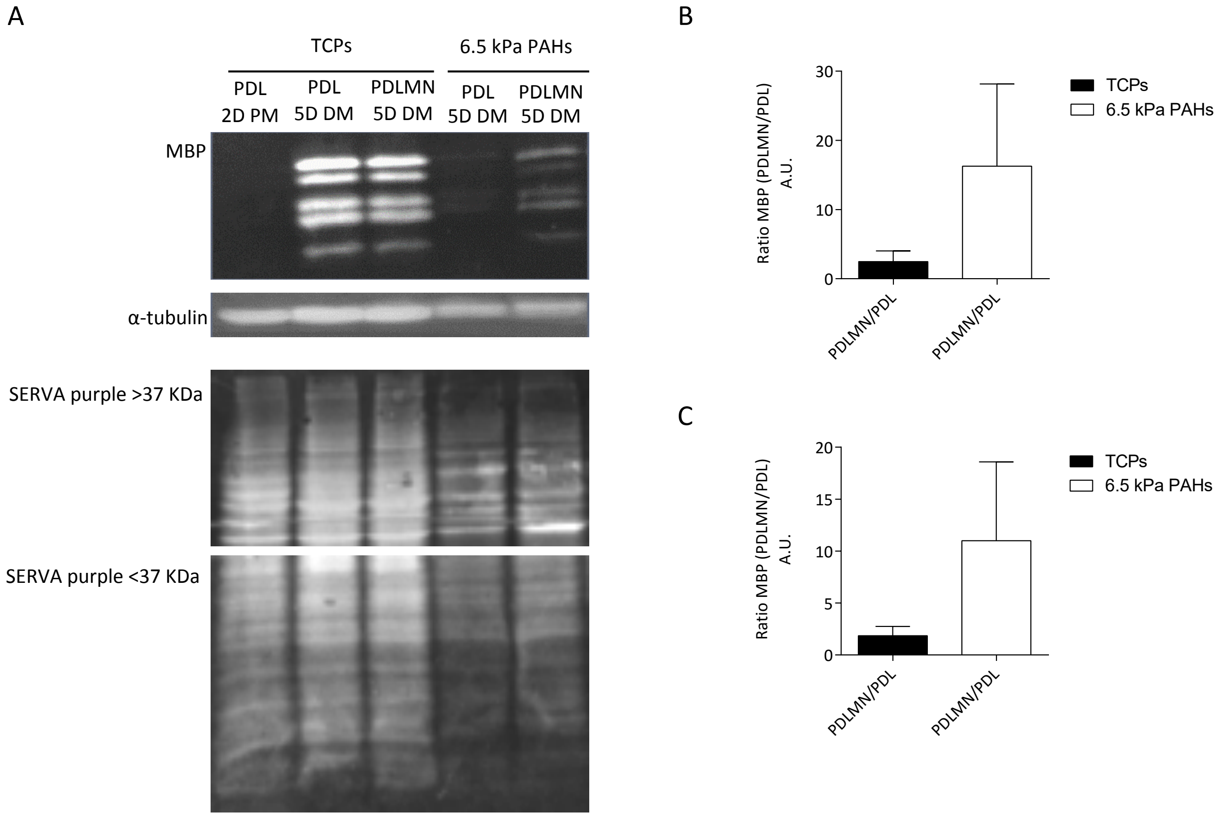

Figure S2 – Assessment of the expression of MBP by western-blot analysis. *(A)* Representative image of western-blot analysis using antibodies against MBP and α-tubulin (as loading control) and total protein staining using SERVA purple reagent. Total protein extracts were obtained from primary rat oligodendrocyte precursor cells (OPCs) in culture on tissue culture polystyrene (TCPs) coated with poly-D-lysine (PDL) in presence of proliferation medium for 2 days (2D PM) or cultured for 5 days in presence of differentiation medium (5D DM) on TCPS or 6.5 kPa PAHs coated/functionalized with PDL or a mixture of PDL and merosin (PDLMN), as indicated. *(B, C)* Ratio of MBP expression by oligodendrocytes differentiated for 5 days (using DM) on substrates (TCPs or 6.5 kPa PAHs, as indicated) coated/functionalized with PDL/merosin relative to PDL alone. α-tubulin expression *(B)* or total protein—using SERVA purple—*(C)* was used as reference to normalize data. Data represent mean ± SEM of 3 independent experiments.

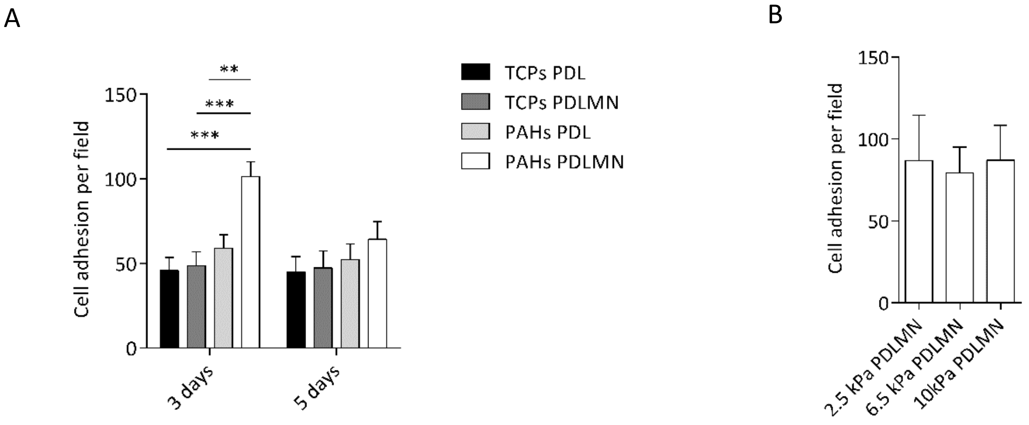

Figure S3 – Primary OPC adhesion on distinct platforms. *(A)* Number of adherent cells per field on TCPs or 6.5 kPa PAHs coated/functionalized with PDL or PDLMN. *(B)* Cell adhesion after 5 days of differentiation on substrates with distinct degrees of stiffness (2.5, 6.5 and 10 kPa). The nuclei (stained with DAPI) were counted using Image J software. In detail, nuclei from at least six fields per independent experiment (at least three) were selected using the "Threshold" tool and the image was converted to black and white (8-bit) and the nuclei were counted using the command "Analyse Particles". Statistical analysis was performed by two-way ANOVA followed by Bonferroni post-test using the software GraphPad Prism 6. Data represent mean ± SEM of at least 3 independent experiments. Statistically significant differences between substrate and coating/ functionalization conditions were represented using connectors (** $p<0.01$, ***$p<0.001$).

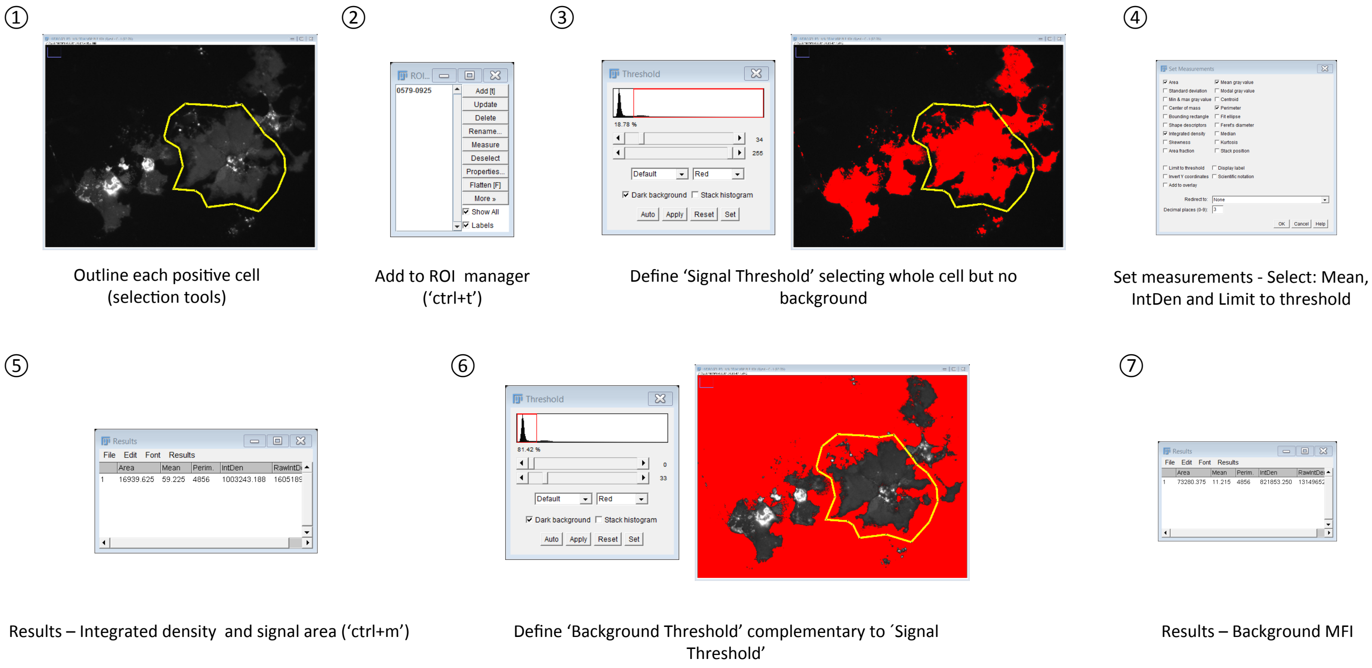

Corrected total cell fluorescence (CTCF) formula:

CTCF= Integrated density – (cell area x mean fluorescence intensity background)

Figure S4 – Step-by-step determination of CTCF by image analysis and quantification of fluorescence microscopy images using Image J software.

Supplementary Table 1 – List of antibodies (and respective dilutions) used for immunocytochemistry (ICC) and western-blot (WB) analysis.

Antibody	Dilution	Brand	Cat. no	Clone
Rat anti-MBP	1:200 (ICC)<br>1:1000 (WB)	Abcam	ab7349	12
Mouse anti-PLP	1:500	Millipore	MAB388	PLPC1
Rabbit anti-Olig2	1:500	Millipore	AB9610	N/A
Mouse anti-α-tubulin	1:1,500 (ICC/WB)	Sigma	T6199	DM1A
Alexa Fluor 488 donkey anti-mouse IgG (H+L)	1:200	Life Technologies	A-21202	N/A
Alexa Fluor 568 goat anti-rat IgG (H+L)	1:200	Life Technologies	A-11077	N/A
Alexa Fluor 568 goat anti-rabbit IgG (H+L)	1:200	Life Technologies	A-11036	N/A
Alkaline phosphatase anti-rat IgG	1:2,500	Santa Cruz Biotechnology	SC-2960	N/A
Alkaline phosphatase anti-mouse IgG (H+L)	1:10,000	Jackson ImmunoResearch laboratories, Inc	115-055-062	N/A
